# Socioecological correlates of perceived cooking skills among Spanish adolescents: the EHDLA study

**DOI:** 10.3389/fpubh.2025.1562110

**Published:** 2025-05-20

**Authors:** Carlos Hermosa-Bosano, José Francisco López-Gil

**Affiliations:** ^1^Well-being, Health and Society Research Group, Universidad de Las Américas, Quito, Ecuador; ^2^Department of Communication and Education, Universidad Loyola Andalucía, Seville, Spain; ^3^Vicerrectoría de Investigación y Postgrado, Universidad de Los Lagos, Osorno, Chile

**Keywords:** cooking, cooking skills, culinary competence, food agency, food literacy

## Abstract

**Background:**

Individuals' perceptions of their cooking skills have been associated with healthier eating patterns. This study examines the socioecological factors associated with adolescents' cooking skills perceptions within a Spanish context. Key factors analyzed included sex, age, immigrant status, socioeconomic status, parental education, family structure, household size, schooling type, and area of residence.

**Methods:**

This research used data from adolescents aged 12–17 who took part of the Eating Healthy and Daily Life Activities (EHDLA) study (Region of Murcia, Spain). A chi-squared test and generalized linear models with binomial distribution were used to examine associations.

**Results:**

Perceptions of cooking skills varied among adolescents; 16.3% rated their cooking skills as very adequate. Perceived cooking skills were significantly associated with sex, socioeconomic status (SES), and type of schooling. Female adolescents had over twice the odds of perceiving their cooking skills as very adequate compared to males [odds ratio (OR) = 2.05, 95% confidence interval (CI): 1.40–3.03, *p* < 0.001]. Adolescents from medium and high SES backgrounds were significantly more likely to report very adequate cooking skills compared to those from low SES backgrounds (OR = 2.17, 95% CI: 1.20–4.13, *p* = 0.013; and OR = 3.57, 95% CI: 1.88–7.08, *p* < 0.001, respectively). Furthermore, attending a private school (with public funds) was associated with lower odds of perceiving cooking skills as very adequate compared to attending a public school (OR = 0.44, 95% CI: 0.24–0.76, *p* = 0.005).

**Conclusions:**

The findings from this study suggest the importance of attending the needs of specific groups such as male adolescents, those from lower SES backgrounds and those in publicly funded private schools. In addition, our results suggest the need to question the gender norms traditionally associated to cooking. Our results can help design interventions that enhance cooking skills among adolescents. These interventions can foster healthier eating habits and ultimately reduce diet-related conditions in adolescence and later in adulthood.

## 1 Introduction

Non-communicable diseases, such as cancer, diabetes, and cardiovascular conditions, have emerged as critical public health challenges in recent decades (1). Previous research has established links between diet quality and these illnesses (2, 3). Evidence further suggests that healthy eating patterns during childhood and adolescence may provide protective effects against disease development later in life (4). The factors associated with diet quality are diverse and operate at multiple levels. At the structural level, food accessibility and affordability, household dynamics, and income levels play a crucial role (5, 6). At the individual level, personal food preferences, attitudes toward healthy eating, among others, shape dietary behaviors (7). As research has evolved, there has been an increasing recognition of cooking and food preparation skills as potentially modifiable factors that contribute to healthier dietary habits, making them a valuable area of focus for nutrition and public health interventions.

Cooking, or the ability to prepare meals using fresh and raw ingredients, is a complex behavior that occurs within broader social, physical, and economic environments that shape an individual's capacity to acquire and apply specific cooking skills (8). The concept of food agency encompasses a range of skills, strategies, and cognitive abilities that enable individuals to make and execute food-related decisions effectively in their contexts (9). Viewing cooking through the concept of food agency allows for a deeper understanding of how individuals interact with their food environments and make choices that promote healthier eating habits. Developing food agency is crucial, as it empowers individuals to navigate their food environments, even when faced with the availability of more convenient or less healthy options, such as ultra-processed foods (9).

The complexity of cooking makes it challenging for researchers to define and assess the array of skills, abilities, and behaviors involved in this task. Traditional assessments of cooking skills have defined them as mechanical and physical techniques necessary for meal preparation, such as chopping, mixing, or heating (10). However, these definitions have been broadened to include other food competencies that extend beyond the kitchen including meal planning, ingredient selection, budgeting, food safety, storage practices, and nutritional knowledge (11, 12). While both cooking and food skills involve concrete actions, they also encompass cognitive and practical elements, such as understanding meal preparation sequences and following recipes (13). Perceptual elements, such as an individual's confidence and motivation, are also important, potentially influencing the likelihood of engaging in cooking and food preparation activities (13). In this context, perceived cooking skills reflect how individuals evaluate their culinary abilities, which can significantly affect their cooking behavior and dietary choices.

Previous studies have found associations between cooking skills with healthier eating patterns, particularly vegetable and fruit consumption and the ability to recognize beneficial food choices (14, 15). Conversely, limited cooking skills could impede healthy eating, potentially contributing to overweight and obesity, particularly among low-income populations (16, 17). In addition, those with low cooking skills may tend to gravitate toward convenience and pre-prepared foods (18). These convenience foods are typically high in calories, saturated fats, and sodium, which are linked to chronic diseases, inflammatory processes, disruptions in the gut microbiome, among other health issues (19, 20). This dependency on pre-prepared meals creates a problematic cycle as increased reliance to convenience foods may decrease cooking motivation and the weakening of existing culinary skills (21).

From a socioecological perspective, cooking skills have been associated to multiple demographic and socioeconomic factors. Directing efforts to understand the broader context of cooking skills among adolescents is necessary as this life stage is crucial for development and the acquisition of healthy eating behaviors (22, 23). Previous studies have shown that women exhibit higher cooking proficiency and confidence across all age groups, from adolescence to adulthood (16, 24). Regarding age, studies suggest potential cohort effects, with younger generations perceiving higher cooking skills (10, 25). Educational attainment and socioeconomic status also play a role, as individuals with higher educational and socioeconomic levels tend to report higher perceptions of their cooking skills (21).

While existing research has explored some factors, important gaps remain in understanding the full range of socioecological factors that shape adolescents' cooking skills. The literature review suggests that several factors including family structure, type of schooling, and area of residence remain understudied. Expanding the available knowledge of these correlates could deepen our understanding of the factors that shape adolescents' current nutritional quality and, potentially, their long-term dietary habits (26). Additionally, examining these correlates can inform the development of interventions that are sensitive to the ecological environments in which adolescents live. Such interventions may have the potential to prevent obesity and other diet-related diseases by equipping adolescents with the skills and confidence needed to establish healthy cooking practices (15).

Based on the above, the aim of this study was twofold: first, to determine the adolescent's self-perceptions of their cooking skills in a sample of Spanish adolescents; and second, to analyze the socioecological factors associated with these perceptions. These factors included sex, age, immigrant status, socioeconomic status, parents' educational level, family structure, number of people at home, type of schooling, and area of residence. Understanding the socioecological factors that influence these skills could help design programs tailored to the unique needs of different adolescent groups. Enhancing cooking skills in adolescents may lead to healthier eating habits, reduced risk of diet-related diseases, and better health outcomes in adulthood.

## 2 Methods

### 2.1 Study design and population

This study is based on a secondary cross-sectional analysis of data from the Eating Healthy and Daily Life Activities (EHDLA) study. The EHDLA study is a research project designed to obtain data on excess weight and its potential sociodemographic, environmental, lifestyle, health-related, cognitive, and psychological correlates among adolescents aged 12–17 years from all the three secondary schools at *Valle de Ricote*, in the Region of Murcia, Spain. In Spain, adolescents aged 12–17 years are typically enrolled in compulsory secondary education (*Educación Secundaria Obligatoria*, ESO) and post-compulsory secondary education (*Bachillerato*). The ESO includes students from 12 to 16 years old, while those aged 16 and 17 may be enrolled in either the final year of ESO or in *Bachillerato*, depending on their academic progression. The detailed methodology of the EHDLA study has been previously outlined by López-Gil (27). For the present study, we used a subsample of participants with complete data on the variables of interest to examine the associations between sociodemographic factors and perceived cooking skills. Data collection was conducted during Physical Education classes in the 2021/2022 academic year, a period in which there were no strict Coronavirus Disease 2019 (COVID-19) lockdowns in Spain. However, public health measures remained in place in schools, including rotating student attendance and the use of “bubble groups.” As a result, data collection was delayed but was ultimately completed following the planned protocol. While these contextual factors may have influenced certain behavioral variables (e.g., physical activity, screen time), we consider it unlikely that they significantly affected the perception of cooking skills or sociodemographic variables. The initial sample consisted of 1,378 adolescents (49.3% males). While the full sample was used for descriptive analyses, only participants with complete data on the variables of interest (*n* = 903) were included in the generalized linear model (GLM) to examine correlates of cooking skills.

Participation in this study required written consent from parents or legal guardians of the selected teenagers. Both parents and participants were provided with an informative document detailing the study's objectives, planned assessments, and surveys. Additionally, the adolescents gave their own consent to participate. The study was conducted in compliance with the guidelines set forth in the Helsinki Declaration. Ethical approval for the study was granted by the Bioethics Committee of the University of Murcia (ID 2218/2018) and the Ethics Committee of the Albacete University Hospital Complex and the Albacete Integrated Care Management (ID 2021-85).

### 2.2 Instruments

#### 2.2.1 Sociodemographic factors

All available sociodemographic variables collected in the EHDLA dataset were included in the multivariable model to ensure a comprehensive analysis and to minimize the risk of omitted variable bias. Age was calculated by asking participants for their birth date. For analytical purposes, participants were also grouped into two age categories: (a) 12–14 years and (b) 15–17 years. Sex was self-reported by participants using binary biological categories (male/female). Students were classified as immigrants if they met at least one of the following criteria: (a) having an immigrant parent, (b) being born outside of Spain, or (c) having at least one parent from another country. Socioeconomic status (SES) was assessed using the Family Affluence Scale (FAS-III), which includes six items: (a) family car ownership (0 = no, 1 = yes, one, 2 = yes, two or more), (b) having their own bedroom (0 = no, 1 = yes), (c) the number of family-owned computers (0 = none, 1 = one, 2 = two, 3 = more than two), (d) the number of bathrooms at home (0 = none, 1 = one, 2 = two, 3 = more than two, (e) owning a dishwasher (0 = no, 1 = yes), and (f) the frequency of family vacations outside of Spain in the past year (0 = not at all, 1 = once, 2 = twice, 3 = more than twice). The total FAS-III score ranges from 0 to 13 points, and participants were categorized into three SES levels: (a) low SES (0–2 points), (b) medium SES (3–5 points), (c) high SES (≥6 points). Additionally, participants were asked to identify the educational level of their father, mother, or legal guardian, with the following options: (a) incomplete primary education, (b) complete primary education, (c) incomplete secondary education, (d) complete secondary education, (e) incomplete higher education, and (f) complete higher education. Adolescents' type of schooling was categorized as either (a) public or (b) publicly funded private schools (also known in Spain as “*centros concertados*”). Both types are state-regulated, but “*centros concertados*” are privately managed and partially financed with public funds. These latter indicators (i.e., educational level and type of schooling) were used for descriptive and exploratory purposes, and were not combined into a composite SES. Adolescents were also asked to report on the number of people at home and identified their family structure, with the options being: (a) two-parent family, (b) blended/compound family, (c) same-sex family, (d) single-parent family (father), (e) single-parent family (mother), (f) extended family, or (g) adoptive family. For analysis, families were classified into four groups: (a) nuclear, (b) single-parent, (c) extended, and (d) diverse. Area of residence was classified as (a) urban (more than 5000 inhabitants) or (b) rural (5000 inhabitants or less).

#### 2.2.2 Perceptions of cooking skills

Perceived adequacy of cooking skills was evaluated by asking participants: “How adequate are your cooking skills?”. The response options included: (a) very adequate, (b) adequate, (c) inadequate, or (d) very inadequate. This single-item measure was selected for its practicality in school-based data collection and has been previously used in adolescent populations to assess perceived culinary competence (26). While not formally validated, its use in prior research and ease of interpretation support its applicability in large-scale studies. For analyses, these responses were grouped into two categories: (a) “very adequate” (including very adequate) and “not very adequate” (including adequate, inadequate and very inadequate). For this study, only responses rated as “very adequate” were considered as indicating a high level of perceived competence. This dichotomization was conceptually justified by our interest in distinguishing adolescents with the highest perceived competence. From a statistical standpoint, preliminary analyses confirmed that combining the three lower categories improved the distributional balance of the binary outcome and enhanced model fit in the GLM. This strategy also allowed for clearer interpretation of the odds ratios in relation to a well-defined outcome category.

### 2.3 Statistical analysis

For categorical variables, frequencies (*n*) and percentages (%) were described. The chi-squared test was used to examine the associations between sociodemographic factors and the perception of cooking skills. Subsequently, a GLM with binomial distribution was employed to further analyze the sociodemographic factors linked to the perception of cooking skills. Odds ratios (ORs) for having very adequate cooking skills, along with their 95% confidence intervals (CI), were estimated for each sociodemographic factor. For the multivariable analysis, we included only participants with complete data on all variables of interest (*n* = 903). This listwise deletion approach was chosen to maintain analytical consistency across all model predictors and avoid the complexity of handling multiple patterns of missing data. While we acknowledge that this strategy may reduce sample size and potentially introduce selection bias, it ensures that each odds ratio is estimated from the same subset of observations, improving model stability and interpretability. All statistical analyses were performed using R statistical software (version 4.3.2) developed by the R Core team in Vienna, Austria, together with RStudio (version 2023.12.1 + 402) from Posit in Boston, Massachusetts, U.S.A. A *p*-value < 0.05 was considered significant for all statistical analyses.

## 3 Results

In Table 1, the perception of cooking skills among the study participants varied, with 16.8% reporting very inadequate skills, 23.3% inadequate skills, 43.6% adequate skills, and 16.3% very adequate skills.

**Table 1:** Descriptive data of the study participants.

**Variable**	***N* = 1378^a^**
**Age group**	
12–14 years	751 (54.5)
15-17y	627 (45.5)
**Sex**	
Males	680 (49.3)
Females	698 (50.7)
**SES**	
Low SES	243 (21.9)
Medium SES	585 (52.7)
High SES	283 (25.5)
*Missing*	267
**Number of siblings**	
None	122 (11.0)
One	585 (52.7)
Two or more	404 (36.4)
*Missing*	267
**Immigrant status**	
Native	836 (75.2)
Immigrant	275 (24.8)
*Missing*	267
**Number of people at home**	
Two or less	186 (16.7)
Three	782 (70.4)
Four or more	143 (12.9)
*Missing*	267
**Race/ethnicity**	
Caucasian	924 (83.2)
Non-Caucasian	187 (16.8)
*Missing*	267
**Type of schooling**	
Public	1,123 (81.5)
Private with public funds	255 (18.5)
**Parental education (mother)**	
Non-university education	800 (73.2)
University education	293 (26.8)
*Missing*	285
**Parental education (father)**	
Non-university education	828 (78.4)
Univerity education	228 (21.6)
**Type of family**	
Nuclear	902 (81.2)
Single-parent	73 (6.6)
Extended	37 (3.3)
Diverse	99 (8.9)
*Missing*	267
**Area of residence**	
Urban	823 (74.1)
Rural	283 (25.9)
*Missing*	267
**Perception of cooking skills**	
Very inadequate	167 (16.8)
Inadequate	232 (23.3)
Adequate	434 (43.6)
Very adequate	162 (16.3)
*Missing*	383

^a^Number (%).

SES, socioeconomic status.

Table 2 shows that sex, socioeconomic status (SES), and type of schooling were significantly associated with the perception of cooking skills among adolescents (*p* < 0.001 for all). A higher proportion of female adolescents reported their cooking skills as very adequate (20.6%) compared to males (11.4%). Likewise, very adequate cooking skills were more frequently reported among individuals with high (23.4%) and medium SES (16.0%) compared to those with low SES (8.9%). In addition, adolescents attending public schools more often reported very adequate cooking skills (18.7%) than those in private schools with public funding (8.3%). In addition, the proportion of adolescents reporting very adequate cooking skills was 14.8% among those living with two or fewer people, 18.2% among those living with three people, and 8.9% among those living with four or more individuals (*p* = 0.041).

**Table 2:** Sociodemographic correlates according to the perception of cooking skills among adolescents.

**Variable**	**Non-very adequate *N* = 754**	**Very adequate *N* = 149**	***p*-value^†^**
**Age group**			
12–14y	483 (85.0%)	85 (15.0%)	0.105
15–17y	271 (80.9%)	64 (19.1%)	
**Sex**			
Boys	358 (88.6%)	46 (11.4%)	< 0.001
Girls	396 (79.4%)	103 (20.6%)	
**SES status**			
Low SES	164 (91.1%)	16 (8.9%)	< 0.001
Medium SES	410 (84.0%)	78 (16.0%)	
High SES	180 (76.6%)	55 (23.4%)	
**Number of siblings**			
None	83 (83.8%)	16 (16.2%)	0.218
One	400 (81.6%)	90 (18.4%)	
Two or more	271 (86.3%)	43 (13.7)	
**Immigrant status**			
Two or more	578 (83.2%)	117 (16.8%)	0.621
Native	176 (84.6%)	32 (15.4%)	
**Number of people at home**			
Two or less	127 (85.2%)	22 (14.8%)	0.041
Three	525 (81.8%)	117 (18.2%)	
Four or more	102 (91.1%)	10 (8.9%)	
**Race/ethnicity**			
Caucasian	640 (83.7%)	125 (16.3%)	0.759
Non-Caucasian	114 (82.6%)	24 (17.4%)	
**Type of schooling**			
Public	578 (81.3%)	133 (18.7%)	< 0.001
Private with public funds	176 (91.7%)	16 (8.3%)	
**Parental education (mother)**			
Non-university education	544 (84.5%)	100 (15.5%)	0.214
University education	210 (81.1%)	49 (18.9%)	
**Parental education (father)**			
Non-university education	585 (83.5%)	116 (16.5%)	0.943
University education	169 (83.7%)	33 (16.3%)	
**Type of family**			
Nuclear	645 (83.3%)	129 (16.7%)	0.666
Single-parent	26 (81.2%)	6 (18.8%)	
Extended	19 (79.2%)	5 (20.8%)	
Diverse	64 (87.7%)	9 (12.3%)	
**Area of residence**			
Urban	560 (84.2%)	105 (15.8%)	0.336
Rural	194 (81.5%)	44 (18.5%)	

Data expressed as number (percentage). This analysis includes a total of 995 adolescents with complete data on the variables of interest. SES, socioeconomic status. ^†^Pearson's Chi-squared test; Fisher's exact test.

Table 3 presents the results of the generalized linear model. Perceived cooking skills among adolescents were significantly associated with sex, socioeconomic status (SES), and type of schooling. Female adolescents had over twice the odds of perceiving their cooking skills as very adequate compared to males (OR = 2.05, 95% CI: 1.40–3.03, *p* < 0.001). Adolescents from medium and high SES backgrounds were significantly more likely to report very adequate cooking skills compared to those from low SES backgrounds (OR = 2.17, 95% CI: 1.20–4.13, *p* = 0.013; and OR = 3.57, 95% CI: 1.88–7.08, *p* < 0.001, respectively). Additionally, attending a private school (with public funds) was associated with lower odds of perceiving cooking skills as very adequate compared to attending a public school (OR = 0.44, 95% CI: 0.24–0.76, *p* = 0.005). No other predictors showed significant associations.

**Table 3:** Generalized linear model with binomial distribution determining the sociodemographic correlates of a very adequate perception of cooking skills among adolescents.

**Predictors**	**OR**	**95% CI**	***p*-value**
**Age group**			
12–14 years	Reference		
15–17 years	1.23	0.84, 1.78	0.282
**Sex**			
Males	Reference		
Females	2.05	1.40, 3.03	< 0.001
**SES**			
Low SES	Reference		
Medium SES	2.17	1.20, 4.13	0.013
High SES	3.57	1.88, 7.08	< 0.001
**Number of siblings**			
None	Reference		
One	0.98	0.47, 2.10	0.963
Two or more	0.76	0.35, 1.70	0.490
**Immigrant status**			
Native	Reference		
Immigrant	1.08	0.57, 1.98	0.813
**Number of people at home**			
Two or less	Reference		
Three	1.21	0.64, 2.39	0.570
Four or more	0.59	0.23, 1.48	0.265
**Race/ethnicity**			
Caucasian	Reference		
Non-Caucasian	1.58	0.79, 3.20	0.198
**Type of schooling**			
Public	Reference		
Private with public funds	0.44	0.24, 0.76	0.005
**Parental education (mother)**			
Non-university education	Reference		
University education	1.30	0.84, 2.00	0.240
**Parental education (father)**			
Non-university education	Reference		
University education	0.77	0.47, 1.23	0.286
**Type of family**			
Nuclear	Reference		
Non-nuclear	1.15	0.64, 1.99	0.183
**Area of residence**			
Urban	Reference		
Rural	1.23	0.81, 1.85	0.313

CI, confidence interval; OR, odds ratio; SES, socioeconomic status.

This analysis includes a total of 903 adolescents with complete data on the variables of interest.

## 4 Discussion

In this study, 16.3% of participants perceived their cooking skills as very adequate. Sex, socioeconomic status (SES), and type of schooling emerged as significant predictors of these perceptions. Female adolescents, those from higher SES backgrounds, and students attending public schools were more likely to report greater confidence in their cooking abilities. These findings highlight how different demographic, and social factors relate to adolescents' self-perceived skills and may inform the development of targeted interventions aimed at promoting cooking skills among youth in the Spanish context.

While our study measured sex as reported by participants, it is likely that underlying gender norms and socialization processes better explain adolescents' perceptions of their cooking skills. Historically, food preparation has been assigned to women, and cooking has been associated with femininity (28, 29). From an early age, girls are more frequently encouraged and often expected to engage in food preparation at home, whereas boys typically receive less exposure and reinforcement in this domain (30). These socially constructed expectations are generationally transmitted and socially reinforced. Research in different cultural contexts, for example, has shown that mothers are typically the primary figures responsible for cooking and for teaching these skills to other family members, reinforcing the association between women and domestic food roles and the transmission of gender-related schemas (10, 31, 32). Consequently, girls may be more frequently exposed to messages suggesting that they should enjoy and excel at cooking, leading them to higher exposure, greater familiarity with kitchen tools, and increased confidence compared to their male counterparts. In addition, societal messaging around health, body image, and food tends to target girls more intensively, which may increase their interest in cooking as a means of exercising control over their food choices and health (33).

It should be noted, however, that there are studies that contradict our findings. Some research have suggested shifts among younger generations, with men increasingly perceiving cooking as vital daily life skill (34). Studies, for example, have found that the percentage of men cooking among the working and upper classes have increased in some European countries and the US (28, 35). Also studies have suggested broader cultural changes, such as the growing visibility of men in culinary roles and their representation across different media platforms (35, 36). Beyond individual and socioeconomic influences, cooking skills are also shaped by cultural norms, traditions, and values. In many contexts, cooking is not merely a practical activity but a culturally embedded behavior that reflects social roles, gender expectations, and intergenerational transmission of knowledge. These cultural patterns can influence not only who cooks and how, but also how individuals perceive their own cooking abilities. Therefore, health literacy interventions aiming to enhance cooking skills among adolescents must be culturally responsive. Such programs should consider local food practices, gender norms, and family dynamics, and incorporate culturally relevant examples and teaching strategies to increase engagement and effectiveness. By doing so, they could better support the development of food agency and long-term healthy eating behaviors.

Regarding SES, there are several possible reasons why SES emerged as a predictor of cooking skills. Higher SES individuals may have increased access to resources such as healthier food options, material resources (e.g., better quality ingredients, cooking equipment, cooking books) and spaces that may facilitate cooking learning and thus, may improve their perceptions regarding their abilities (37). Enhanced educational opportunities may further contribute to improved food literacy and cooking confidence (38). Furthermore, it is likely that parents in higher SES households hold higher cooking skills, potentially modeling healthier eating behaviors and encouraging adolescents to participate in cooking activities from a young age (39).

SES-related differences in cooking's cultural perception may also explain our results. For individuals from privileged backgrounds, cooking may operate not only as a practical ability but also as a marker of social class and cultural status (40). According to cultural capital theory, individuals from higher SES backgrounds may develop a series of skills, knowledges, and preferences that distinguish them from other social groups and this may have an impact on their food choices, and possibly their cooking skills (40). Moreover, higher SES individuals are potentially more exposed to social media content that frames cooking as a creative and rewarding activity (25).

Another explanation may lie in household food consumption patterns. Some studies have found a positive association between higher SES and a greater frequency of home-cooked meals, while individuals from lower SES groups are more likely to consume take-away or fast foods (41). However, other research suggests that higher-income individuals may also purchase more pre-prepared convenience foods, potentially reducing the necessity to cook and complicating the relationship between SES and their perceived cooking competence (11). These contradictory findings highlight the need for further research to disentangle the ways in which access to cooking education, exposure to convenience foods, and cultural attitudes toward cooking shape both perceived and actual cooking skills across different socioeconomic groups.

Regarding type of schooling, adolescents attending publicly funded private schools (i.e., “centros concertados”) were less likely to perceive themselves as highly competent in cooking. One possible explanation is that these schools are more likely to provide school lunch programs (especially in this region), reducing adolescents' exposure to meal preparation at home and, consequently, their opportunity to develop cooking-related confidence. Moreover, families who enroll their children in such schools may place greater emphasis on academic performance or structured extracurricular activities, with less involvement in domestic tasks like cooking. This limited exposure may contribute to lower perceptions of culinary competence. Previous studies have found that greater reliance on prepared foods or institutional meals is associated with lower cooking confidence and reduced skill development (21, 37, 41). Additionally, cultural factors may play a role; in some social groups, cooking may be perceived less as a necessary life skill and more as a low-prestige or outsourced activity, in line with the concept of cultural capital (40). Thus, these findings suggest the importance of considering both structural and cultural factors when addressing skill development in adolescents across different educational settings.

There are several limitations that need to be acknowledged. First, the cross-sectional design of the study inherently limits our capacity to establish causal relationships between variables. Moreover, in this study we assessed cooking skills using a single-item measure that asked participants to rate how adequate they believed their cooking skills were. While this approach provides a quick insight into self-perceived ability and has been previously used (26), it certainly does not capture the multidimensional nature of cooking skills (12). Previous studies have called upon the lack of consistent measures capable of assessing components of such a complex construct (12). Additionally, much work remains to be done in improving the validation of questionnaires in this field. A review by Texeira and colleagues (42) emphasized the limited information on the psychometric properties of existing instruments for assessing culinary skills, suggesting a critical need for better validated tools. This limitation suggests that our findings might not fully represent adolescents' actual culinary abilities and may actually reflect overestimations of their skills. This tendency to overestimate their confidence may be related to response biases where individuals report higher confidence in their abilities despite limited practical experience or the need to exercise particular skills (5). Future research would benefit from the use of multidimensional, validated instruments to better assess culinary competence, which would enhance construct validity and strengthen the overall robustness of findings. Moreover, the listwise deletion applied during the multivariable analysis is another limitation. Out of the 1,378 participants in the initial sample, only 903 had complete data on all relevant variables and were thus included in the GLM. This represents a 34.5% reduction in the sample size. Although the remaining sample was still sufficiently large and demographically diverse to support robust analyses, this loss may introduce a risk of selection bias. We compared descriptive and bivariate results between the full and analytical samples and found consistent patterns (data not shown), suggesting that the overall findings are likely generalizable. Nevertheless, caution is warranted when interpreting the multivariable results, and future studies should consider strategies such as multiple imputation to minimize data loss. Finally, this study was conducted in a specific Region of Spain (Murcia), and as such, the findings may not be generalizable to all Spanish adolescents or to adolescents in other countries. Cultural factors, regional socioeconomic patterns, and local educational systems may influence both cooking behaviors and perceptions of cooking skills. Therefore, caution is advised when extrapolating these results beyond this regional context.

Based on this, this study could have benefited from employing more comprehensive assessments that include objective measures of cooking proficiency (e.g., standardized cooking tasks or skill demonstrations) alongside self-reported perceptions. Such an approach would enable a deeper understanding of perceived and actual cooking skills and how these dimensions may vary by demographic factors, such as sex, socioeconomic status, or cultural background. Furthermore, we acknowledge that adolescents may not always accurately report their parents' social status, but prior studies have shown acceptable reliability of these self-reports in large-scale school-based research (43, 44). Finally, although the classification of immigrant status follows definitions commonly used in Spanish population-based studies (45, 46), we acknowledge that this approach does not capture self-perceived ethnicity or cultural identity, which could provide a more nuanced understanding of adolescents' backgrounds.

Despite its limitations, this study has several strengths, including its focus on adolescents. Addressing this age group is particularly important, as it provides valuable insights that can inform interventions aimed at enhancing culinary competencies (22). Given that female adolescents reported greater confidence in their cooking abilities, educational programs should aim to engage male adolescents actively in food preparation, challenging traditional gender norms that may limit their participation in domestic tasks. Similarly, the association between higher SES and greater perceived cooking competence indicates a need to address disparities in access to cooking education and resources. Adolescents from lower SES backgrounds may benefit from initiatives that provide access to cooking facilities, affordable ingredients, and mentorship opportunities. In addition, our findings highlight the importance of incorporating cooking and food literacy programs into the curricula of publicly funded private schools, where students may have fewer opportunities to engage in meal preparation due to the availability of school lunch programs. Introducing hands-on culinary education in these settings could help build adolescents' cooking confidence and promote healthier eating habits. Overall, the adoption of a socioecological framework to examine various correlates of cooking perceptions is a notable strength due to the complex nature of these competencies. Further studying these factors can lead to the development of tailored interventions, designed specifically to address the needs from adolescents living in lower socioeconomic conditions.

## 5 Conclusions

This study identified a significant association between sex, socioeconomic status and type of schooling regarding their perceptions of cooking skills. This finding highlights the importance of tailoring interventions to address the specific needs of these populations (18). Future research should delve deeper into the social and cultural factors that shape adolescents' cooking skills, as well as the barriers that hinder their development. Additionally, it is essential to continue exploring contemporary perceptions of cooking and how these perceptions may have an impact in adolescents' nutritional and health-related behaviors and trajectories over time.

## Data Availability

The raw data supporting the conclusions of this article will be made available by the authors upon reasonable request.
